# Social Big-Data Analysis of Particulate Matter, Health, and Society

**DOI:** 10.3390/ijerph16193607

**Published:** 2019-09-26

**Authors:** Juyoung Song, Tae Min Song

**Affiliations:** 1 Department of Administration of Justice, Pennsylvania State University, Schuylkill Haven, PA 17972, USA; 2 Department of Health Management, Sahmyook University, Seoul 01795, Korea; tmsong01@hanmail.net

**Keywords:** Social Big-Data Analysis, Particulate Matter, health, South Korea

## Abstract

The study collected particulate matter (PM)-related documents in Korea and classified main keywords related to particulate matter, health, and social problems using text and opinion mining. The study attempted to present a prediction model for important causes related to particulate matter by using social big-data analysis. Topics related to particulate matter were collected from online (online news sites, blogs, cafés, social network services, and bulletin boards) from 1 January 2015, to 31 May 2016, and 226,977 text documents were included in the analysis. The present study applied machine-learning analysis technique to forecast the risk of particulate matter. Emotions related to particulate matter were found to be 65.4% negative, 7.7% neutral, and 27.0% positive. Intelligent services that can detect early and prevent unknown crisis situations of particulate matter may be possible if risk factors of particulate matter are predicted through the linkage of the machine-learning prediction model.

## 1. Introduction

Air pollution due to rapid industrialization and urbanization has become the fourth-biggest threat to global human health following hypertension, dietary habits, and smoking [1]. The World Health Organization (WHO) estimates that premature death due to air pollution is reaching seven million, by which the proportion of death due to outdoor air pollution includes ischemic heart disease at 40%, stroke at 40%, chronic obstructive pulmonary disease at 11%, lung cancer at 6%, and acute lower respiratory infections in children at 3% [2]. In addition, extreme weather phenomena and air pollution influence communicable diseases due to water, food, insect vectors, and rodents [3]. The major causes of air pollution are particulate matter (PM_2.5_, PM_10_), sulfur dioxide (SO_2_), and nitrogen oxides (NO_2_), which produce particulate matter (PM) by directly polluting the air or change into secondary pollutants through chemical reactions in the atmosphere [1]. In particular, since the International Agency for Research on Cancer (IARC) classified PM as a Group-1 carcinogen [4], interest in the effects of PMs on health is growing [5]. Particulate matter has been reported to increase the risk of respiratory-related diseases such as asthma exacerbation and chronic obstructive pulmonary disease, as well as cardiovascular diseases such as irregular heartbeats, vascular dysfunction, and arrhythmia [6]. It is also reportedly related to acute and chronic premature death [7].

South Korea set the standard for Total Suspended Particles (TSP) in the “Framework Act on Environmental Policy” in 1991 before adding PM_10_ and PM_2.5_ to the Act in 1993 and 2011, respectively. The number of deaths due to the air pollution in South Korea is estimated to have been 11,944 (24 deaths per 100,000 population) in 2008 (http://apps.who). Generally, air pollution level is represented by daily or annual mass concentration (µg/m^3^) of particulate matter or ultrafine particles in the air. The WHO recommends daily and annual averages of ultrafine particles (PM_10_) equal to or less than 50 µg/m^3^ and 20 µg/m^3^, respectively. Because South Korea has higher particulate-matter concentration than other countries, damage from particulate-matter exposure is quite high [8]. The PM_10_ pollution concentration in South Korea was 51–61 µg/m^3^ during 2001–2006, but, due to the implementation of an air-quality management plan in the Seoul metropolitan area (2005–2014), it has been decreasing since 2007. The improvement in air quality has, however, stagnated recently [9]. Especially in the case of the capital area and big cities, energy use is high due to the concentration of economic activities [10], and, when winter begins, a high concentration of air pollution is observed due to long-distance transportation of highly concentrated particulate matter along with smog from China [11]. In 2013, 42% of air-pollutant emission facilities and 45% of registered vehicles were concentrated in the Seoul metropolitan area and its vicinities. The PM_10_ concentration in Seoul in 2011 was 2.1 times higher than Tokyo, 1.6 times higher than Paris, and 1.4 times higher than London [12].

Health concerns are increasing because air pollutants can be transported in from northeastern China along with yellow sand. The analysis of the effects of yellow sand on deaths in Seoul from 1995 to 1998 showed that death risks increased by 1.7% during the yellow sand period. Among the population aged 65 years or older, however, death risks increased by 2.2% [13]. The analysis of the relationship between the concentration of air pollutants in seven metropolitan cities and hospitalization data from 2001 to 2005 showed significant correlations between air pollution and both cardiovascular and respiratory diseases. A comparison between persons aged 65 or older and those under 65 for the relationship with different diseases showed that those aged 65 or older were significantly affected by air pollution in the form of arrhythmia, heart disease, heart failure, ischemic heart disease, asthma, and pneumonia [14]. In addition to the elderly, children can also suffer adverse health effects such as asthma because they have a higher respiratory rate per body weight and have characteristic behavior and life patterns [5]. The components of particulate matter can damage agricultural produce, soil, and aquatic organisms; failure of traffic and navigation, as well as the breakdown of outdoor machines and building facilities, can also occur [9]. Particulate matter is composed of suspended particles from construction sites and vehicles; primary pollutants, which include from boiler combustion and vehicles; and secondary pollutants produced by the interaction between various pollutants in the air. Because the particulate matter transported from China also accounts for a large part, investigation of particulate-matter sources is difficult [15]. As concerns for the adverse effects of particulate matter on health and the environment deepen, the demand for responsive policies at the government level is intensifying, but effective government policies are difficult to implement due to the lack of diversified research on the causes of particulate-matter pollution. Many developed countries forecast particulate matter based on statistical models and let people use the information in their daily lives. Groups vulnerable to particulate matter such as the elderly, children, and bronchial patients are actively using the forecast information about particulate matter in their lives [15]. In the study titled, “Associations between Long-Term Particulate-Matter Exposure and Adult Renal Function in the Taipei Metropolis”, the generalized linear and logistic regression models were used to estimate the associations between PM exposure and renal function [16]. In the study titled, “Characterization of Fine Particulate Matter and Associations between Particulate Chemical Constituents and Mortality in Seoul, Korea”, by using Poisson generalized linear model, found that link PM_2.5_ constituents with mortality and have implications for policy making on sources of PM_2.5_ and on the relevance of PM_2.5_ health studies from other areas to this region [17]. Cobourn developed a nonlinear regression model using two years of air-quality and weather data-sets of the three large Chinese cities of Beijing, Nanjing, and Guangzhou [18]. Brian et al. developed the National Air-Quality Forecast Capability (NAQFC) and provide next-day ozone concentration forecasts for the adjacent areas of the United States [19]. Giri et al. analyzed the relationship between PM_10_ concentration and weather conditions in the Kathmandu area, Nepal, and found that seasons greatly affect PM_10_ concentration and air quality [20]. Furthermore, Seinfeld and Pandis proved that the diffusion of pollutants is determined by atmospheric conditions such as wind speed, temperature, and insolation [21].

In addition, as the interest in artificial intelligence increases, various research studies predicting particulate matter using machine learning have been conducted at home and abroad. Papanastasiou et al. collected air pollution and meteorological data to predict PM_10_ concentration in the Volos area, Greece, through a neural network and a regression model, and proved that air pollution is deeply related to meteorological elements [22]. Chaloulakou et al. proved that the forecast error by a neural network model is smaller than that of a linear regression model using temperature, relative humidity, wind direction, and wind speed as explanatory variables to predict the daily average concentration of particulate matter smaller than 10 microns [23]. Barai et al. collected 15-year (1985–1999) annual average data of VOC, NO_x_, CO, SO_2_, PM_10_, and PM_2.5_ provided on the US EPA website and daily average data of RPMA (Respiratory Particulate Matter Average) from 3 July 2000, to 20 August 2001, provided on the website of the Tata Energy Research Institute, and analyzed it, finding that a neural network model is appropriate if it has a large-scale training data set [24]. Ghazi and Khadir collected PM_10_ data along with temperature data such as wind speed, humidity, and temperature from 1 January 2003, to 12 December 2004, and developed an RNN (Recurrent Neural Network) model [25]. Ong et al. used wind direction, wind speed, temperature, amount of sunshine, humidity, and precipitation data of 52 cities in Japan to predict PM_2.5_, and developed a Deep Recurrent Neural Network (DRNN) model with a reinforced pre-training method using an autoencoder in the process of learning [26]. Hooyberghs et al. developed a neural network model that predicts the daily average PM_10_ concentration in Belgium and found that the changes in daily average PM_10_ concentration in Belgian urban areas is mainly influenced by weather conditions such as previous PM_10_ concentration, wind speed, temperature, the amount of clouds, and wind direction, but less influenced by anthropogenic factors such as mechanical friction of fossil fuels, smelting, and combustion [27]. Koo et al. constructed forecast models for the same day and next day using air pollution measurement data, observation data from ground and high-rise weather stations, and weather forecast data through neural network analysis, regression analysis, and decision-making model analysis [15]. Lee et al. proposed a model forecasting the daily average and daily maximum particulate matter pollution using regression analysis, neural network analysis, and support vector regression analysis [28].

Regarding the research related to East Asian region, Mahajan, Chen, and Tsai examined PM_2.5_ forecasting using comparative analysis of prediction performance for the additive version of Holt-Winters method, autoregressive integrated moving average (ARIMA) model and neural network autoregressive model (NNAM) in Taiwan [29]. Luo, Yang, Huang, Mahajan, and Chen proposed to build a real-time forecasting system with high accuracy and deploy it in Taiwan. They forecast method called Adaptive Iterative Forecast (AIF) based on the trend of historical data [30]. Mei, Li, Fan, Zhu, and Dyer proposed a complementary approach to monitor Air-Quality Index (AQI) by using machine-learning models to estimate AQI from social media posts in China [31]. Similarly, Wang, Paul, and Dredze examined the value of Chinese social media for monitoring air-quality trends and related public perceptions and response [32].

Recently, the Social Network Service (SNS) has been emerging as a communication tool for emergency and crisis response as its influence increases. The SNS is a space where concerns over anxiety about various social phenomena occurring in everyday life can be heard and their forms understood. The results of social media issue analysis conducted by the Ministry of Security and Public Administration showed that the public responded sensitively in that anxious Tweets increased when a particulate-matter watch was issued and rapidly changed to relieved Tweets of relief when the watch was lifted. In addition, various methods for coping with the discomfort and dissatisfaction with particulate matter were shown when the concentration of yellow sand was the highest in mid-March 2014 [29]. As such, the analysis of the relationship among emotional expressions or the causes and symptoms people feel related to particulate matter allows the detection of significant patterns along with danger signs of particulate matter, and it can have positive effects on government policies toward particulate matter by preventing the danger of exposure to particulate matter and timely responses.

The present study collected PM-related documents mentioned in all online channels from which documents can be collected in South Korea and classified main keywords related to particulate matter using text and opinion mining and attempted to present a prediction model for important causes related to particulate matter.

## 2. Materials and Methods

### 2.1. Research Targets

The present study used social big data collected through the Internet such as online news sites, blogs, cafés, social network services, and bulletin boards. The present analysis defined social big data as text-based web documents (buzz) collectible through a total of 166 online channels including 146 online news sites, four blogs (Naver, Daum, Nate, Tistory), two cafés (Naver, Daum), one SNS (Twitter), and 13 bulletin boards (such as Naver “Jishik-iN,” NateTalk, NatePann, and DaumAgora). Topics related to particulate matter were collected every hour regardless of the day of the week, weekends, and holidays from the applicable channels from 1 January 2015, to 31 May 2016, and 226,977 text documents that mentioned causes and diseases related to particulate matter out of a total of 587,099 collected documents were included in the analysis. A crawler was used to collect social big data for the present study, and text-mining techniques were used to classify topics. Particulate matter topics used to collect all relevant documents were “particulate matter, ultrafine particles, yellow sand, smog, atmospheric pollution, and air pollution,” and documents were collected after removing documents using stop words such as “smog wrinkles, contaminated children, smog films, and smog work,” which were unrelated to particulate matter, during the collection period. The main purpose of social big-data analysis is to examine complex social and environmental issued based on the various opinion of large amount of online data. By conducting social big-data analysis, we can predict more accurately to find risk factors or protective factors of particulate matter.

### 2.2. Research Instruments

Text documents collected in relation to particulate matter were encoded as standardized data through the text-mining process as follows.

#### 2.2.1. Emotions Related to Particulate Matter

Emotions related to particulate matter, which were dependent variables of the present study, were defined according to classifications through text-mining in which positive emotions (such as happy, neat, cool, pleasant, healing, possible, strong recommendation, refreshing, joy, recommendation, overcome, positive, expectation, clean, fortunate, great, satisfaction, harmless, relaxing, belief, crisp, invigorating, fresh, relief, safety, stability, agree, solve, cure, and comfort) were defined as positive, and negative emotions (such as bad, danger, exceed the standard, watch, disaster level, worst, serious, stuffy, inconvenience, terror, anxiety, depression, fatigue, pain, fatal, care, damage, stifling, worry, high risk, bafflement, extreme, horrible, failure, perplexed, catastrophe, great confusion, fear, problem, opposition, resistance, emission, neglect, denial, dissatisfaction, helplessness, vicious circle, crisis, disaster, calamity, caution, abstinence, warning, and concern) were defined as negative. Emotional dictionary includes positive feelings (e.g., clean, safe) and negative feeling (e.g., unpleasant, depressed) in the online document. The emotional dictionary was developed by SK Telecom Korea’s leading communication company. In addition, if positive and negative attitudes were equal, they were defined as neutral.

#### 2.2.2. Causes Related to Particulate Matter

Causes related to particulate matter were defined with 17 keywords classified in the subject analysis, which include “dust, yellow sand, PM_10_, powder, tobacco, grilling, influenced China, PM_2.5_, air pollution, ozone, smog, pollutant, carcinogen, fossil fuel, bacteria, exhaust gas, and chemical substance.”

#### 2.2.3. Diseases Related to Particulate Matter

Diseases related to particulate matter were defined with seven keywords, “common cold, lung disease, cardiac disorder, cerebrovascular disease, hypertension, depression, death disease,” which were classified through text-mining.

### 2.3. Analysis Methods

The present study applied machine-learning analysis technique to forecast the risk of particulate matter. Representative machine-learning algorithms used in the present study were random forest, decision-tree analysis, and multilayer neural network. In addition, to determine the relationships among the independent variables that influence the risk of particulate matter, association analysis was carried out. An a priori principle algorithm was used for the association analysis. Social big data and data mining is based on the causal relationship between the emotional effects of PM. The research finding could provide the risk factors and protective factor on the PM issue. The ROC (Receiver Operating Characteristic) curve and AUC (Area under the Curve) were used for the evaluation of machine-learning models. IBM SPSS 24.0 (SPSS Inc., Chicago, IL, USA) was used for the decision-tree analysis, and R 3.4.2 (R Foundation for Statistical Computing, Vienna, Austria) was used for the random forest, multilayer neural network analysis, association analysis, and model evaluation.

## 3. Results

### 3.1. Status of Online Documents on Particulate Matter

Status of online documents related to particulate matter is shown in Table 1. Emotions related to particulate matter were found to be 65.4% negative, 7.7% neutral, and 27.0% positive. Causes related to particulate matter were in the following order from the highest to the lowest: yellow sand (25.9%), smog (11.9%), PM_2.5_ (9.7%), air pollution (9.1%), dust (7.5%), and pollutants (6.3%). Diseases related to particulate matter were in the following order: common colds (45.9%), lung disease (18.6%), cardiac disorder (10.4%), cerebrovascular disease (8.0%), death disease (7.4%), depression (5.0%), and hypertension (4.7%), from the highest to the lowest.

### 3.2. Factors Affecting the Risk of Particulate Matter

**Figure 1:**
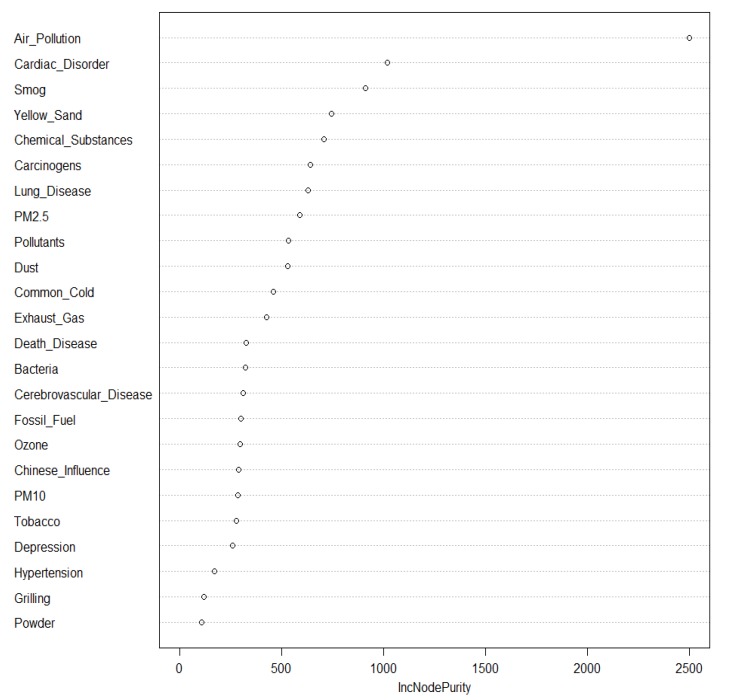
Random Forest Model of Cause-and-Disease Factor.

The results of the analysis of main factors influencing the emotions related to particulate matter (Non-negative, Negative) using a random forest model are presented in Figure 1. The figure showing the importance (IncNodePurity) of the random forest model indicates that the main factor that has the greatest influence on emotions related to particulate matter (an important factor that classifies non-negative and negative emotions) is “Air Pollution.” It is followed by cardiac disorder, smog, yellow sand, chemical substance, carcinogen, lung disease, PM_2.5_, and pollutants.

**Figure 2:**
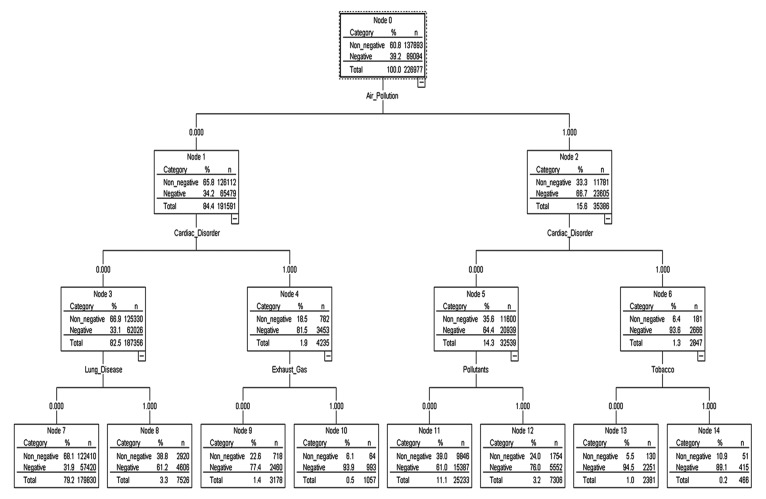
Decision-Tree Model of Cause-and-Disease Factor.

**Figure 3:**
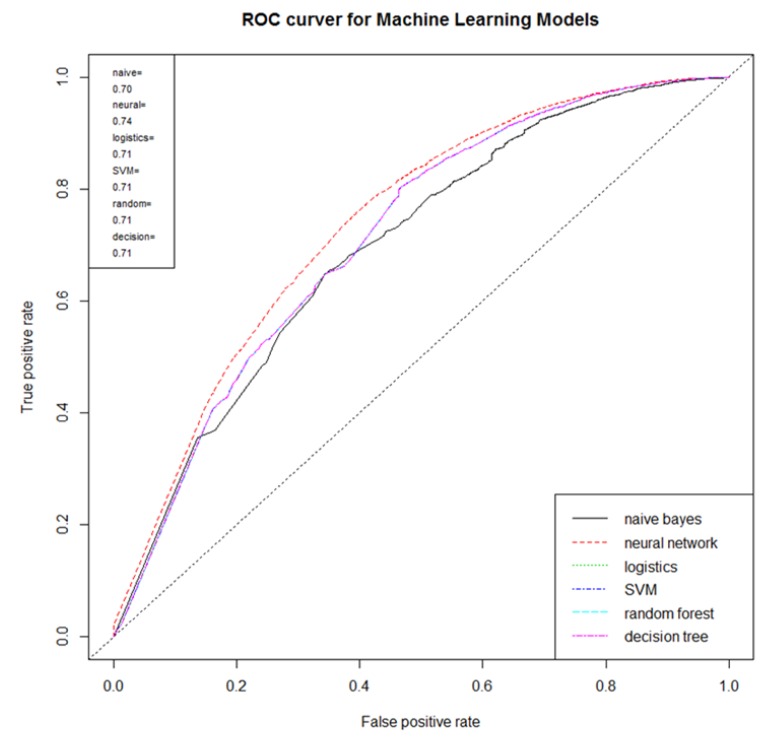
Random Forest Model of Cause-and-Disease Factor.

The decision-tree model for the prediction of the risk factor of particulate matter is shown in Figure 2. The root tree at the top of the tree structure shows the frequency of the dependent variable without the predictor variables (independent variables) entered. The emotion ratio for particulate matter of the root node was 39.2% negative and 60.8% non-negative. Since the cause-and-disease factor at the top under the root node is the factor that has the greatest influence (highly relevant) on the dependent variable, the influence of the “air pollution” factor was found to be the largest, i.e., negative emotions about particulate matter increased from 39.2% to 66.7% if an online document had the air pollution factor in it. Negative emotions about particulate matter increased from 66.7% to 93.6% when air pollution and cardiac disorder factors were in the document. To develop a prediction model for the risk of particulate matter, 226,977 text documents that mentioned causes and diseases related to particulate matter were used as a learning data set. Training data and test data were sampled 50:50 from learning data to develop a prediction model. Analysis of machine learning on the cause of particulate matter and the risk of diseases showed that the neural network (AUC = 0.74) performed best (Figure 3). 

**Figure 4:**
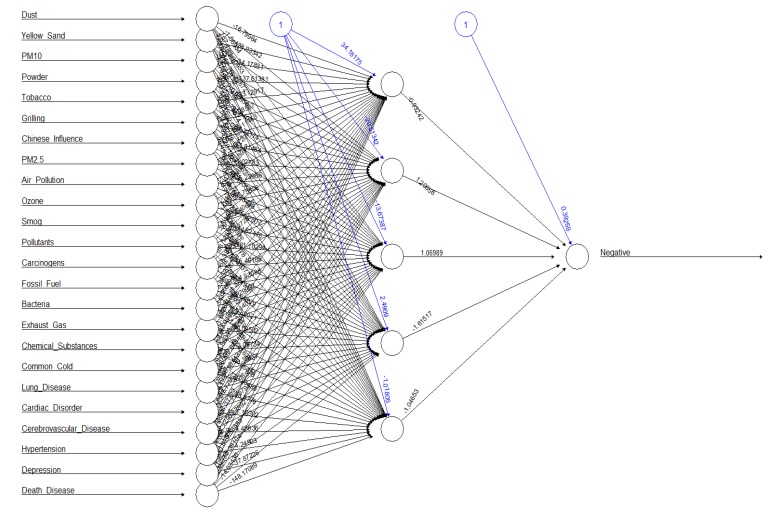
Particulate-Matter Cause-and-Disease Risk Multilayer Neural Network Prediction Model.

A multilayer neural network model using 17 causes related to particulate matter (dust, yellow sand, PM_10_, powder, tobacco, grilling, influenced China, PM_2.5_, air pollution, ozone, smog, pollutant, carcinogen, fossil fuel, bacteria, exhaust gas, and chemical substance) and seven diseases (common cold, lung disease, cardiac disorder, cerebrovascular disease, hypertension, depression, death disease) as the input layer, and five hidden layers and risk (Negative) as one output layer is shown in Figure 4. The overall risk of causes-and-disease factors predicted by the multilayer neural network model was 39.35%. The risk of each factor was in the following order: smog (8.21%), influenced China (5.19%), carcinogen (4.29%), pollutant (3.83%), death disease (2.37%), yellow sand (1.94%), tobacco (1.88%), fossil fuel (1.64%), ozone (1.42%), cardiac disorder (1.22%), exhaust gas (0.95%), bacteria (0.87%), chemical substance (0.8%), lung disease (0.79%), PM_10_ (0.79%), common cold (0.56%), cerebrovascular disease (0.52%), grilling (0.49%), air pollution (0.48%), depression (0.35%), powder (0.31%), hypertension (0.23%), PM_2.5_ (0.14%), and dust (0.08%), from the highest to the lowest.

**Figure 5:**
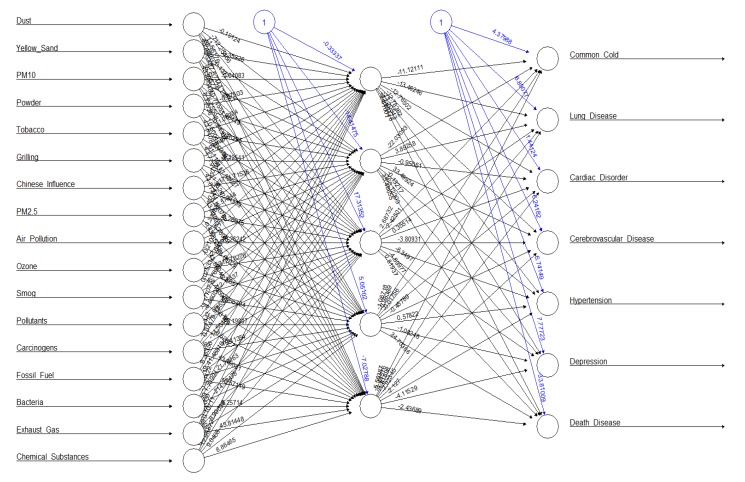
Disease Multilayer Neural Network Prediction Model for Causes of Particulate Matter.

A multilayer neural network model using 17 causes related to particulate matter as the input layer, and five hidden layers and seven diseases as the output layer, is shown in Figure 5. The accuracy of the neural network model for the prediction of diseases caused by particulate matter was in the following order: the common cold (10.49%), lung disease (5.19%), cardiac disorder (2.80%), cerebrovascular disease (2.14%), death disease (1.88%), hypertension (1.13%), and depression (0.71%), from the highest to the lowest.

Association analysis in social big-data analysis is performed to discover the relationships between two or more words included in an online document. The present study analyzed association rules between the causes of particulate matter and disease factors as shown in Table 2. The results showed that the association between the four factors {pollutant, carcinogen, common cold} ≥ {lung disease} was 0.011 support, 0.647 confidence, and 11.64 lift, and the same rule is seen in 2471 documents. It indicates that when “pollutant, carcinogen, common cold” factors are mentioned in an online document, the probability of the document mentioning lung disease is 64.7%, and the probability of the document mentioning lung disease is 11.6 times higher than in a document that does not mention “pollutants, carcinogens, common cold.”

**Figure 6:**
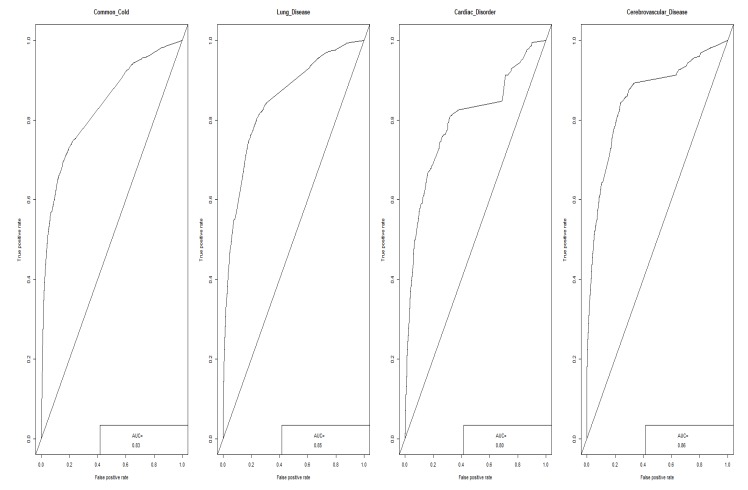

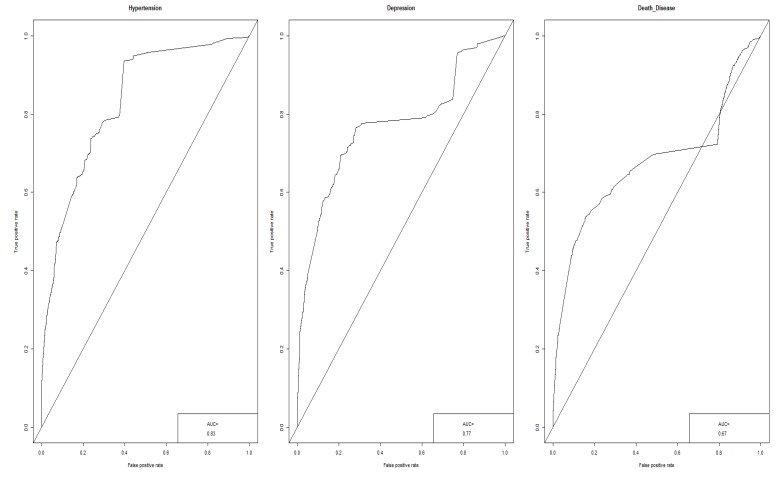
Performance of Machine Learning for Predicting Disease Causes of Particulate Matter.

The risk prediction of the disease-prediction neural network model of the causes of particulate matter showed a similar trend to the particulate matter (PM_10_, PM_2.5_) forecast by the Korea Meteorological Administration (Figure 6).

## 4. Discussion

The purpose of the present study was to develop a risk prediction model for particulate matter by collecting PM-related documents mentioned in all online channels from which documents can be collected in South Korea and using them as machine-learning data. The summary and implications of the present study are as follows.

First, emotions related to particulate matter were found to be 65.4% negative, 7.7% neutral, and 27.0% positive. The finding is similar to that of the Public Attitudes towards the Environment—2016 Survey [33] in which 68.6% of the respondents worried about the of particulate matter and ultrafine particles on health. The direct causes among the causes related to particulate matter are in the following order: exhaust gas, the influence of China, fossil fuels, and tobacco, from the highest to the lowest. The finding is similar to that of the survey of the Korean Federation for Environmental Movement [34], which found the causes of particulate matter were the influence of the neighbor countries such as China, exhaust gas including diesel cars, and coal-fired electrical power plants. Disease factors related to particulate matter were in the following order: common cold, lung disease, heart disease, and cerebrovascular disease, from the highest to the lowest. These are similar to the findings of particulate matter threats investigated by the Korean Environment Institution (KEI) [33], which found threats in the following order: cough, rhinitis, and sinusitis, asthma, acute and chronic bronchitis, atopic dermatitis, dizziness and headaches, cardiovascular disease, and cerebrovascular disease, from the highest to the lowest.

Second, the factors that have the greatest effect on emotions related to particulate matter in the random forest model were in the following order: air pollution, heart disease, smog, yellow sand, chemicals, carcinogens, lung disease, PM_2.5_, and pollutants, from the highest to the lowest. In the decision-tree model, negative emotions about particulate matter increased by about 2.4 times from 39.2% to 93.6% when air pollution and cardiac disorder factors were in the document than when the two factors were not present. This signifies that if cardiac disorder is present due to air pollution when particulate matter is mentioned in an online document, the risk of particulate matter increases 2.4 times.

Third, the performance of multilayer neural networks was found to be best for the evaluation of machine learning for the cause of particulate matter and risk prediction of diseases. This supports previous studies that reported superior prediction accuracy of the neural network in the development of a particulate matter prediction model using machine learning [22,23,24,25,27].

Fourth, the overall risk of causes-and-disease factors predicted by the multilayer neural network model was 39.35%. The risk of the causes of particulate matter by factor was in the following order: smog, influence of China, carcinogens, pollutants, yellow sand, tobacco, fossil fuel, ozone, exhaust gas, bacteria, chemical substance, PM_10_, grilling, air pollution, powder, PM_2.5_, and dust. The risk of disease by particulate matter by factor was in the order of death disease, cardiac disorder, lung disease, common cold, cerebrovascular disease, depression, and hypertension, from the highest to the lowest. In the multilayer neural network model for the prediction of the influence of the causes of particulate matter on diseases, the prediction probability of particulate matter and related causative factors was in the following order: common cold, lung disease, cardiac disorder, cerebrovascular disease, death disease, hypertension, and depression, from the highest to the lowest.

Fifth, in association analysis between the cause of particulate matter and disease factors, the probability of mentioning lung disease increased by about 11.6 times when “pollutant, carcinogen, and common cold” factors are mentioned, and the common cold is found to be interconnected with yellow sand, dust, pollutants, lung disease, carcinogens, cardiac disorders, and bacteria. Lastly, the risk prediction of the disease-prediction neural network model on the cause of particulate matter showed a similar trend to the particulate-matter (PM_10_, PM_2.5_) forecast by the Korea Meteorological Administration.

## 5. Conclusions

The policy implications and conclusions of the findings of the present study are as follows. The implication of the finding is to predict the risk of PM and predict the degree of PM by using the prediction model. The research can provide more accurate weather information related to PM concentration and prevention system can be established.

First, when causes and diseases related to particulate matter are mentioned in online documents, the negative emotions toward particulate matter increase. Accordingly, management as well as promotion measures need to be prepared through accurate diagnosis of the causes of particulate matter for correct public understanding. To that end, the establishment of countermeasures based on the identification of causes through the analysis of various big data of particulate matter and an integrated management system that includes the influence of neighboring countries and the interaction of climate changes appears to be needed.

Second, the prevalence rate of diseases due to particulate matter is serious. Particulate matter has been reported to increase the risk of respiratory-related diseases such as asthma exacerbation and chronic obstructive pulmonary disease, and cardiovascular diseases such as irregular heartbeats, vascular dysfunction, and arrhythmia [6]. It also has been reported to be related to acute and chronic premature death. In the multilayer neural network analysis of the present study, the prediction probability is in the following order: common cold, lung disease, cardiac disorder, cerebrovascular disease, and death disease, from the highest to the lowest. Because information is searched or shared online when a person is infected with a disease such as the common cold due to various particulate-matter causes, as a means to overcome the disease, verified information can be provided online in advance by predicting related diseases due to particulate matter using the machine-learning prediction model developed in the present study.

Third, as the existing particulate-matter prediction models use the data measured by organizations such as the Korea Meteorological Administration, the neural network model was also found to be superior for prediction models using social big data. Since the input variable used as the learning data of the neural network of the present study, however, used words about causes and diseases mentioned in relation to particulate matter, and positive and negative emotional words in relation to particulate matter as output variables, it can be less accurate than the existing measurements of causes, diseases, and emotional state by the existing operational definitions. Accordingly, the development of an analysis technique that can perform subject analysis of causes, diseases, and emotions at the sentence level appears to be necessary.

Fourth, high-quality learning data with accurate classification are needed to increase the accuracy of the machine-learning model for particulate matter developed in the present study, and to that end, the development of ontology for a terminology system for particulate matter and a dictionary of emotional words is needed.

Fifth, continuous data updates used in the machine-learning model are necessary. In the case of machine-learning models developed through the learning of training data, the actual classification and predicted classification are different when test data are applied. Accordingly, the prediction accuracy of machine-learning models can be improved if training data are learned again after producing high-quality training data by selecting cases for which classification of actual data and predicted data are the same to increase the prediction accuracy rate of the model.

Sixth, the development of glossaries of colloquial or slang words for the causes of particulate matter and diseases and a system that allows collecting such words is necessary since many general consumers do not use professional terminology of the causes of particulate matter and diseases. Lastly, intelligent services that can detect early and prevent unknown crisis of particulate matter may be possible if risk factors of particulate matter are predicted through the linkage of the machine-learning prediction model for particulate matter developed in the present study with weather big data and disease big data.

## Figures and Tables

**Table 1:** Status of online documents related to particulate matter.

Factors	Variables	*N* (%)		Factors	Variables	*N* (%)	
Emotion	Negative	89,084	(65.4)	Cause	Dust	29,258	(7.5)
	Neutral	10,455	(7.7)		Yellow sand	100,545	(25.9)
	Positive	36,745	(27.0)		PM_10_(PM_10_)	5260	(1.4)
	Sub-Total	136,284			Powder	1985	(0.5)
	No expressions of emotion	90,693			Tobacco	6977	(1.8)
	Total	226,977			Grilling	581	(0.1)
Disease	Common cold	31,205	(45.9)		Chinese influence	11,317	(2.9)
	Lung disease	12,650	(18.6)		PM_2.5_(PM_2.5_)	37,529	(9.7)
	Cardiac disorder	7082	(10.4)		Air pollution	35,386	(9.1)
	Cerebrovascular Disease	5419	(8.0)		Ozone	7948	(2.0)
	Hypertension	3166	(4.7)		Smog	46,316	(11.9)
	Depression	3413	(5.0)		Pollutants	24,427	(6.3)
	Death disease	5010	(7.4)		Carcinogens	19,628	(5.0)
	Total	67,945			Fossil fuel	11,081	(2.8)
					Bacteria	14,248	(3.7)
					Exhaust gas	19,989	(5.1)
					Chemical substances	16,344	(4.2)
					Total	388,819	

**Table 2:** Association Rule between Cause-and-Disease Factor.

Rules	Support	Confidence	Lift	Count
{Pollutants,Carcinogens,Common_Cold} ≥ {Lung_Disease}	0.010886566	0.646689348	11.60344729	2471
{Common_Cold,Cardiac_Disorder} ≥ {Lung_Disease}	0.010269763	0.624932976	11.21307605	2331
{Carcinogens,Exhaust_Gas} ≥ {Chemical_Substances}	0.010838103	0.67937034	9.434743122	2460
{Fossil_Fuel,Chemical_Substances} ≥ {Exhaust_Gas}	0.010014231	0.72342457	8.21455494	2273
{Chemical_Substances,Lung_Disease} ≥ {Carcinogens}	0.010040665	0.630254425	7.288223893	2279
{Pollutants,Common_Cold,Lung_Disease} ≥ {Carcinogens}	0.010886566	0.616055846	7.124032395	2471
{Dust,Carcinogens,Common_Cold} ≥ {Pollutants}	0.01085132	0.738530735	6.862467374	2463
{Carcinogens,Common_Cold,Lung_Disease} ≥ {Pollutants}	0.010886566	0.705798343	6.558316231	2471
{Dust,Pollutants,Lung_Disease} => {Common_Cold}	0.01085132	0.894985465	6.509889951	2463
{Chemical_Substances,Common_Cold} ≥ {Pollutants}	0.013538817	0.696351688	6.470537403	3073
{Pollutants,Carcinogens,Lung_Disease} ≥ {Common_Cold}	0.010886566	0.888529306	6.46292954	2471
{Dust,Yellow_Sand,Lung_Disease} ≥ {Common_Cold}	0.010410746	0.886679174	6.449472168	2363
{Dust,Carcinogens} ≥ {Pollutants}	0.022284196	0.691739606	6.427681687	5058
{Lung_Disease,Cardiac_Disorder} ≥ {Common_Cold}	0.010269763	0.865256125	6.293646512	2331
{Dust,Lung_Disease} ≥ {Common_Cold}	0.017213198	0.850457118	6.186002412	3907
{Dust,Chemical_Substances} ≥ {Pollutants}	0.013847218	0.663080169	6.161376652	3143
{Pollutants,Lung_Disease} ≥ {Common_Cold}	0.017671394	0.844065657	6.139512595	4011
{Bacteria,Chemical_Substances} ≥ {Pollutants}	0.010829291	0.656166578	6.097135191	2458
{Yellow_Sand,Carcinogens,Common_Cold} ≥ {Pollutants}	0.010062694	0.645562465	5.998601201	2284
{Bacteria,Lung_Disease} ≥ {Common_Cold}	0.013640149	0.815595364	5.932427138	3096
{Dust,Yellow_Sand,Carcinogens} ≥ {Pollutants}	0.010331443	0.636536374	5.914730276	2345
{Air_Pollution,Lung_Disease} ≥ {Pollutants}	0.010556136	0.632690784	5.878996853	2396
{Dust,Common_Cold,Lung_Disease} ≥ {Pollutants}	0.01085132	0.630406962	5.857775453	2463
{Air_Pollution,Lung_Disease} ≥ {Common_Cold}	0.013234821	0.793240032	5.769820307	3004
{Carcinogens,Common_Cold} ≥ {Pollutants}	0.016834305	0.607279085	5.642869971	3821
{Yellow_Sand,Lung_Disease} ≥ {Common_Cold}	0.018208893	0.739620616	5.379806713	4133
{Carcinogens,Lung_Disease} ≥ {Common_Cold}	0.01542447	0.722451506	5.254923107	3501
{Yellow_Sand,Pollutants,Common_Cold} ≥ {Dust}	0.011657569	0.665995469	5.166643436	2646
{Yellow_Sand,Carcinogens,Common_Cold} ≥ {Dust}	0.010124374	0.649519503	5.038826582	2298
{Chemical_Substances,Lung_Disease} ≥ {Common_Cold}	0.011001115	0.690542035	5.02282197	2497
{Pollutants,Carcinogens,Common_Cold} ≥ {Dust}	0.01085132	0.644595656	5.000628482	2463
{PM_2.5_, Lung_Disease} ≥ {Common_Cold}	0.010732365	0.664303245	4.831967879	2436
{Lung_Disease} ≥ {Common_Cold}	0.036805491	0.660395257	4.803542196	8354
{Pollutants,Common_Cold,Lung_Disease} ≥ {Dust}	0.01085132	0.614061331	4.763750045	2463
{Yellow_Sand,Pollutants,Carcinogens} ≥ {Dust}	0.010331443	0.609724389	4.730105019	2345
{Dust,Yellow_Sand,Carcinogens} ≥ {Common_Cold}	0.010124374	0.623778502	4.537201505	2298
{Pollutants,Exhaust_Gas} ≥ {Air_Pollution}	0.014107156	0.638994213	4.098711056	3202
{PM_10_} ≥ {PM_2.5_}	0.014446398	0.62338403	3.770253326	3279
{Dust,Carcinogens,Common_Cold} ≥ {Yellow_Sand}	0.010124374	0.689055472	1.555519856	2298
{Dust,Pollutants,Common_Cold} ≥ {Yellow_Sand}	0.011657569	0.65140325	1.470521213	2646
